# Temporal Development of Autonomic Dysfunction in Heart Failure: Effects of Age in an Ovine Rapid-pacing Model

**DOI:** 10.1093/gerona/glv217

**Published:** 2015-12-26

**Authors:** Margaux A. Horn, Elizabeth F. Bode, Samantha J. Borland, Graeme J. Kirkwood, Sarah J. Briston, Mark A. Richards, Katharine M. Dibb, Andrew W. Trafford

**Affiliations:** Unit of Cardiac Physiology, Manchester Academic Health Sciences Centre.

**Keywords:** Age-related pathology, Animal model, Cardiovascular disease

## Abstract

Heart failure (HF) is predominantly a disease of older adults and characterized by extensive sympatho-vagal imbalance leading to impaired reflex control of heart rate (HR). However, whether aging influences the development or extent of the autonomic imbalance in HF remains unclear. To address this, we used an ovine model of aging with tachypacing-induced HF to determine whether aging affects the chronotropic and inotropic responses to autonomic stimulation and reduction in heart rate variability (HRV) in HF. We find that aging is associated with increased cardiac dimensions and reduced contractility before the onset of tachypacing, and these differences persist in HF. Additionally, the chronotropic response to β-adrenergic stimulation was markedly attenuated in HF, and this occurred more rapidly in aged animals. By measuring HR during sequential autonomic blockade, our data are consistent with a reduced parasympathetic control of resting HR in aging, with young HF animals having an attenuated sympathetic influence on HR. Time-domain analyses of HR show a reduction in HRV in both young and aged failing animals, although HRV is lowest in aged HF. In conclusion, aging is associated with altered autonomic control and β-adrenergic responsiveness of HR, and these are exacerbated with the development of HF.

In the healthy heart, stimulation of the sympathetic nervous system via β-adrenergic pathways leads to an increase in heart rate (HR), force of contraction, and rate of relaxation. In general, opposing actions are mediated by the parasympathetic nervous system, with vagal stimulation slowing HR and decreasing contractility. Sympatho-vagal imbalance arising as a result of reduced parasympathetic and increased sympathetic activity is a well-characterized feature of heart failure (HF), contributes to the systolic dysfunction associated with HF, and increasing sympatho-vagal imbalance is associated with progression towards the decompensated state (1,2). For example, the increased circulating levels of catecholamines and thus elevated sympathetic drive in HF lead to reduced responsiveness to β-adrenergic stimulation and a worsening of clinical symptoms (3,4). Moreover, targeting HF-associated sympatho-vagal imbalance through chronic vagal nerve stimulation appears to have therapeutic benefit in both experimental and clinical HF (5,6).

There is also evidence suggesting that aging leads to sympatho-vagal imbalance and mis-control of HR with impaired cardiac responsiveness to β-adrenergic stimulation noted (7–10). Furthermore, increased basal levels of circulating catecholamines and decreased β_1_-adrenoreceptor density have also been found with age (11). Aging is also a major risk factor for the development of HF (12). Despite the clear age dependency of HF, there is presently a paucity of information regarding whether alterations to the neurohormonal axis that occur in aged animals with HF develop to the same degree, or with the same time dependency, as that which occurs in young animals with HF. These gaps in the literature exist primarily because the majority of experimental research is carried out using only young animals. In this study, we have used a ventricular tachypacing model of HF in both young and aged sheep to investigate any age dependency in the temporal development of sympatho-vagal imbalance in HF using in vivo techniques on conscious animals.

We find that aging is associated with an increase in resting HR, reduction in heart rate variability (HRV), and a reduced change in cardiac contractility in response to β-adrenergic stimulation. The development of HF increased HR, reduced HRV, attenuated the HR response to β-adrenergic stimulation, and abolished vagal tone in both young and aged sheep. However, the impairment of β-adrenergic responsiveness occurred earlier in aged sheep following induction of HF by tachypacing. Also, during complete pharmacologically mediated autonomic blockade, the intrinsic HR was found to be increased in aging. We conclude therefore that HF leads to reduced catecholamine responsiveness in both the young and aged, but these effects develop more rapidly in the aged.

## Methods

All procedures involving the use of animals were performed in accordance with The United Kingdom Animals (Scientific Procedures) Act, 1986 and European Union Directive 2010/63. Institutional approval was obtained from The University of Manchester Animal Welfare and Ethical Review Board. Furthermore, the study accords with the ARRIVE guidelines (13).

### Experimental Model of Aging and HF

Female Welsh Mountain sheep (*n* = 39; 34.8±1.0kg) aged either 18 months (young, *n* = 23) or 8 years or older (aged, *n* = 16) were loose housed in groups of 4–5, fed ad libitum hay, and maintained on a 12-hour light, dark cycle at 20±2°C. Animals were anesthetized with isoflurane (1%–5% v/v in oxygen), and preoperative analgesia was provided (meloxicom 0.5mg/kg). A single active fixation bipolar IS-1 endocardial pacing lead was placed trans-venously into the right ventricle and secured to a single chamber cardiac pacemaker with software modification (High Rate Pacing Patch, Medtronic Inc). To induce HF, right ventricular tachypacing was initiated at 210 beats per minute (bpm). In two animals, cardiac capture was not immediately consistent at 210 bpm; we therefore paced these animals at 180 bpm for 2–7 days before increasing to 210 bpm for the remainder of the study. On presentation of clinical signs of HF (lethargy, dyspnea), tachypacing was stopped and a final “postpacing” set of in vivo experiments performed. Animals were humanely killed with pentobarbitone sodium (200 mg·kg^−1^) with 10,000 units of heparin administered intravenously, and tissues were harvested for additional studies. For all in vivo assessments of cardiac function and sympatho-vagal balance following initiation of right ventricular tachypacing, pacemakers were switched off (ODO) for 15 minutes before measurements were taken. All in vivo experiments were carried out on conscious, nonsedated, and gently restrained animals.

### Echocardiography and Blood Pressure Measurements

Echocardiograms were analyzed offline using ImageJ (NIH, USA), and 3–20 consecutive cardiac cycles were averaged to obtain measurements. Left ventricular (LV) end diastolic internal diameter (EDID), end systolic internal diameter (ESID), relative wall thickness (RWT), and fractional shortening measurements were all obtained from m-mode images, whereas the fractional area change was measured from short-axis images as we have described previously (4,14–17). Systolic and diastolic blood pressure measurements were recorded by tail cuff plethysmography. Readings were obtained over a 3- to 5-minute period, and the last three consistent values were averaged following European Society of Hypertension (ESH) and European Society of Cardiology (ESC) guidelines (18).

### Measurement of HR and HRV

Surface electrocardiograms (ECGs) were recorded using a five-lead orthogonal configuration, digitized to personal computer (1kHz), and the RR interval and HR were analyzed as described previously using shape recognition algorithms (ECG Auto, EMKA France) (17). Time-domain measures of HRV were quantified as RR interval standard deviation (RRSD) and the coefficient of variance of RRSD (RRSD / mean RR interval).

### Assessment of Temporal Development of Sympathetic Dysfunction

The development of sympathetic dysfunction with tachypacing was monitored weekly with intravenous infusions of the sympathetic agonist dobutamine (5–40 µg·kg^−1^·minute^−1^, Claris Life Sciences, UK). Dobutamine concentrations were increased in twofold increments up to a maximum of 40 μg·kg^−1·^minute^−1^ or until HR exceeded 150 bpm. ECGs were recorded for 5 minutes at each dobutamine dose, and ECG parameters were calculated from the last 60 seconds of each infusion period. Animals were allowed to recover for at least 30 minutes before tachypacing was recommenced. To assess whether sympathetic-mediated enhancement of cardiac contractility was altered with HF and aging, parasternal short-axis echocardiograms were obtained at both baseline and immediately following the final infusion of dobutamine (5 µg·kg^−1^·minute^−1^ in control and 10–40 µg·kg^−1^·minute^−1^ in HF). Measurements were obtained both prior to tachypacing (control) and once HF had developed.

### Assessment of Parasympathetic Influence of HR and HRV

Parasympathetic function in aging and HF was measured using sequential sympathetic (0.5 mg·kg^−1^ propranolol [PPL]; prepared as propranolol hydrochloride in 0.9% NaCl solution, Sigma-Aldrich, UK) and parasympathetic (0.05 mg·kg^−1^ atropine; prepared as atropine sulfate in 0.9% NaCl solution; Sigma-Aldrich) blockade via intravenous bolus with an additional 0.05 mg·kg^−1^·minute^−1^ atropine infused after 1 minute to maintain complete autonomic block (AB) for the duration of the experiment. Measurements were obtained both prior to the start of tachypacing and once HF had developed. To determine whether AB was complete, HR responses to dobutamine (5 µg·kg^−1^·minute^−1^) and acetylcholine (100 nmol·min^−1^; prepared as acetylcholine chloride in 0.9% NaCl; Sigma-Aldrich) were assessed in a subset of animals.

### Quantification of Plasma Noradrenaline

Blood samples (~ 20mL) were collected into K_2_EDTA vacutainer blood collection tubes by jugular venipuncture. Samples were stored on ice prior to centrifugation (4,000*g*, 10 minutes), and plasma were harvested and stored in liquid nitrogen until use. Plasma noradrenaline was quantified using a competitive ELISA according to manufacturer’s instructions (Labor Diagnostika Nord, Germany).

### Statistics

All data are presented as the mean ± *SEM* for *n* animals. All comparisons were made pair-wise on the same animal—that is, control versus HF as well as before and during drug infusions. Differences between groups (age, disease, and drug treatments) were analyzed using either a two-way repeated measures analysis of variance (ANOVA) firstly within each age group (eg, control vs HF; pre and post drug administration) and then between age groups (ie, young control vs aged control) or paired Student’s *t* test (autonomic blockade vs dobutamine or acetylcholine). Data that were not normally distributed were transformed by means appropriate to the spread of data, for example, log_10_ (19). Differences were considered significant when *p* value was less than .05; however, *p* values were adjusted using the Holm-Sidak post hoc test for multiple comparisons using SigmaPlot 11.0. Correlational analysis was performed using GraphPad Prism 6.

## Results

Signs of HF (lethargy and dyspnea) took a median of 35 days to develop (interquartile range = 9) with no age-dependent differences in the duration of tachypacing required to induce HF (*p* = .56). Tachypacing was not associated with any instances of sudden death, and all animals completed the study with none being excluded from subsequent data analysis.

As in our previous studies (4,14–17), aging and tachypacing-induced HF were associated with increased LV dimensions and reduced contractile function (Table 1). Of note, aging by itself was associated with a 21% ± 7% increase in EDID, a 50% ± 15% increase in ESID, and a 15% ± 4% decrease in fractional shortening (all *p* < .01). In both age groups, HF increased (*p* < .001) EDID (young by 55.6% ± 10%; aged by 34.8% ± 6%) and ESID (young by 232% ± 21%; aged by 152% ± 20%) and resulted in a reduction (*p* < .001) in fractional shortening (young by 57% ± 2%; aged by 59% ± 3%). However, ESID and fractional shortening were worst in the aged HF animals (*p* < .05). Finally, HF was associated with a decrease in RWT (*p* < .001; young by 51% ± 5%; aged by 41% ± 5%). Systolic, diastolic, and mean blood pressure were decreased similarly with HF in both young and aged animals (all *p* < .001).

**Table 1.** Summary of Echocardiographic and Hemodynamic Findings in Aging and Heart Failure

	Young		Aged	
	Prepaced	Postpaced	Prepaced	Postpaced
EDID (cm)	2.61±0.10	3.91±0.07***	3.15±0.15**	4.13±0.10^†††^
ESID (cm)	0.92±0.06	2.82±0.10***	1.38±0.11**	3.17±0.12^†††,#^
Fractional shortening	0.67±0.02	0.29±0.02***	0.57±0.02***	0.23±0.02^†††,#^
RWT	0.91±0.08	0.39±0.02***	0.73±0.06*	0.42±0.05^†††^
Fractional area change	0.68±0.02	0.38±0.02***	0.63±0.02	0.36±0.02^†††^
Systolic BP (mmHg)	127±2.36	108±2.27***	133±2.09	114±2.62^†††^
Diastolic BP (mmHg)	79±2.0	67±1.64***	84±2.29	71±2.36^†††^
Mean BP (mmHg)	95±1.96	81±1.70***	100±1.96	85±2.17^†††^

*Notes:* Values are mean ± *SEM* from *n* = 16–18 young, 10–16 aged. Fractional shortening was calculated as (EDID − ESID) / EDID. RWT was calculated as (2 × diastolic wall thickness) / EDID. Fractional area change was calculated as (end diastolic area − end systolic area) / end diastolic area.

BP = blood pressure; EDID = end diastolic internal diameter; ESID = end systolic internal diameter; RWT = relative wall thickness.

*p < .05 vs young prepaced. **p < .01 vs young prepaced. ^***^ *p* < .001 vs young prepaced. ^†††^ *p* < .001 vs old prepaced. ^#^ *p* < .05 vs young postpaced.

### HR, HRV, and PR Interval Are Altered in the Ovine Model of Aging and HF

**Figure 1.**
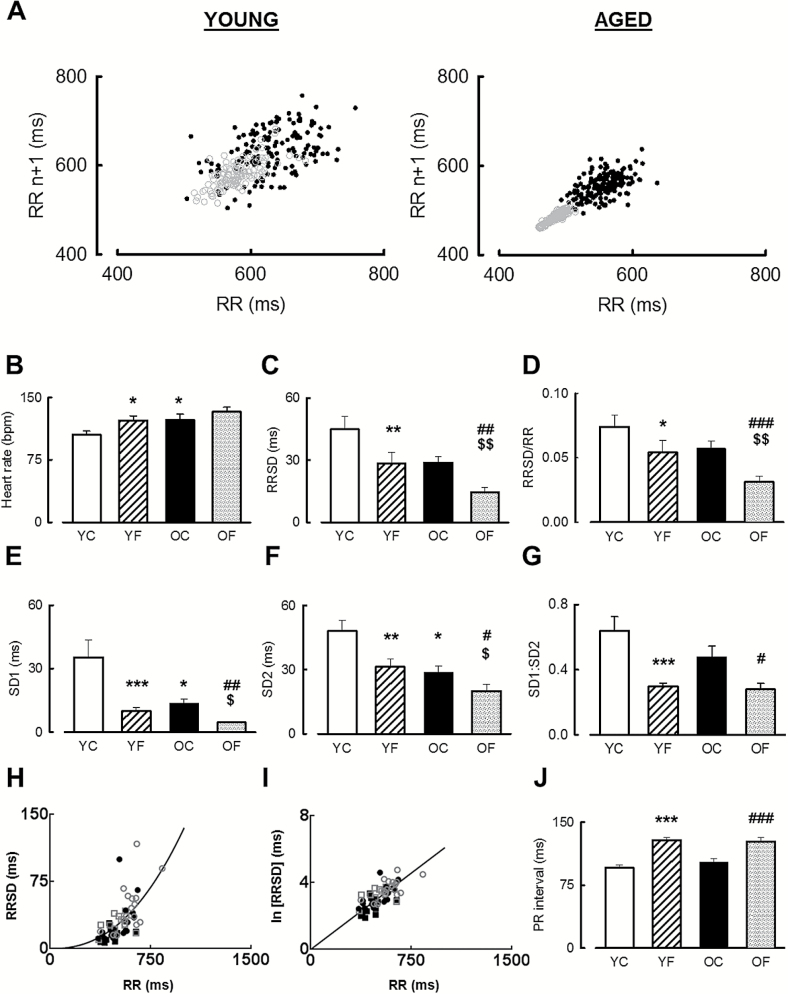
Changes to baseline heart rate heart rate variability and PR interval. (**A**) Representative Poincaré plots of successive RR intervals in young (left) and aged (right) sheep before tachypacing (solid black circles) and in HF (open gray circles). (**B**–**G**) Summary data for young control (YC), young HF (YF), aged control (OC), and aged HF (OF) animals showing (**B**) mean HR, (**C**) RR standard deviation (RRSD), (**D**) coefficient of variance of RRSD, (**E**) instantaneous beat-to-beat HRV (SD1), (**F**) long-term beat-to-beat HRV (SD2), and (**G**) ratio of short- to long-term beat-to-beat HRV (SD1:SD2). (**H**) Mean values of RRSD as a function of RR interval. The solid line through the data is a best-fit regression of the form *y* = *A* + *B* · e^C^ forced through the origin and having exponent ^(C)^ of 2.355. (**I**). **N**atural log RRSD as a function of mean RR interval with the solid line representing a linear regression to the data (*p* < .001). In panels (**H**) and (**I**) symbols denote: ○, YC; ●, YF; □, OC; and ■, OF. (**J**) PR interval summary data. *p < .05 vs YC; **p < .01 vs YC; ***p < .001 vs YC; ^#^ *p* < .05 vs OC; ^##^ *p* < .01 vs OC; ^###^ *p* < .001 vs OC; ^$^ *p* < .05 vs YF; ^$$^ *p* < .01 vs YF. *n* = 13–18 young, 9–11 aged.

Surface five-lead orthogonal ECGs were measured both prior to tachypacing (control) and once HF was evident. Representative Poincaré plots of successive RR intervals from a young and an aged sheep are shown in Figure 1A. Resting HR was higher in aged than in young animals (Figure 1B. young, 105±4 bpm; aged, 123±7 bpm; *p* < .05). HF was associated with an increase in resting HR (122±6 bpm, pacemaker off) only in young animals (*p* < .05).

HRV measured in the time domain as the RRSD (Figure 1C) decreased with the development of HF in both age groups (young: control, 45±6ms; HF, 29±5ms; *p* < .01; aged: control, 29±3ms; HF, 15±2ms; *p* < .01). However, although the observed differences in HRV (RRSD) did not reach significance in aged control animals compared with young control animals (*p* = .094), it was less in aged failing compared with the young failing sheep (*p* < .01) and decreased overall with age (control and HF) compared with young (two-way ANOVA; *p* < .05)

Time-domain measures of HRV such as RRSD are influenced by the underlying HR (20–22), and we also see a strong correlation between RRSD and RR interval in sheep (Figure 1H–I; *p* < .001). We have therefore corrected for the age and HF-dependent changes in HR by normalizing the RRSD values to the mean RR interval in each animal (Figure 1D). We find that the coefficient of variance of the RRSD (20,21) indicates that the reduction in HRV in HF is not simply due to the increased HR (young: control, 0.074±0.009; HF, 0.054±0.009; *p* < .05; Aged: control, 0.057±0.006; HF, 0.031±0.005; *p* < .001). Both measures therefore indicate a reduction of HRV in both young and aged animals with the development of HF. Furthermore, the coefficient of variance of RRSD was decreased overall in control and HF aged animals (two-way ANOVA, *p* < .05)

The final way in which we have assessed HRV in aging and HF is from the short-term (SD1), long-term (SD2), and ratio of short- to long-term variation of successive RR intervals obtained from the Poincaré plots (SD1:SD2; Figure 1E–G). Agreement with the previous assessments of HRV, these measures also show a decrease in HRV with age and HF, with the HF-dependent decrease in SD1:SD2 ratio indicative of loss of parasympathetic influence on HR control being noted in both age groups.

In order to investigate whether conduction remodeling occurs in this model of HF, we quantified PR interval from the ECGs (Figure 1J). HF was associated with an increase in PR interval in both young (128.6±3.3ms vs 95.9±3.2ms) and aged (126.7±4.8ms vs 102.6±3.8ms) sheep (both *p* < .001). However, no difference in PR interval was detected as a result of age (*p* = .57 by two-way repeated measures ANOVA).

### Attenuated Chronotropic Responsiveness to β-Adrenergic Stimulation Occurs Early in the Development of HF by Tachypacing: An Effect Accelerated by Aging

**Figure 2.**
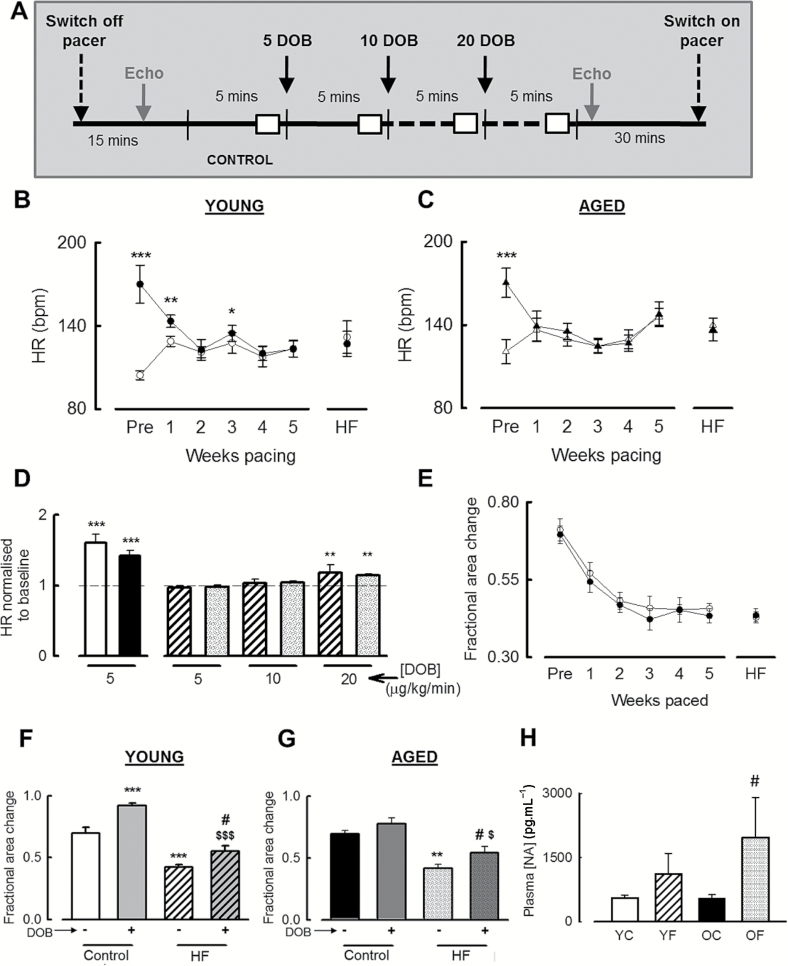
Temporal development of sympathetic dysfunction in heart failure and aging. (**A**) Timeline of experimental protocol used to assess loss of sympathetic responsiveness in HF. White boxes indicate where ECGs were analyzed. (**B**, **C**) Summary data showing mean HR from animals at baseline (open circles) and in response to intravenous infusion of 5 μg·kg^−1^·minute^−1^ dobutamine (DOB, solid circles) in (**B**) young and (**C**) aged sheep. *p < .05; **p < .01; ***p < .001 vs baseline. (**D**) Summary data showing HR normalized to predobutamine infusion HR in young prepaced (open bar), aged prepaced (solid bar), young HF (striped bar), and aged HF (checked bar). **p < .01 vs normalized resting HR (ie, 1); ***p < .001 vs pre-DOB infusion HR. (**E**) LV contractility measured by fractional area change throughout the course of the rapid-pacing protocol in young (solid) and aged (open) sheep. Two-way repeated measures ANOVA detected a significant difference between fractional area change and weeks pacing (*p* < .001). (**F**, **G**). LV contractile function was assessed using 2D short-axis echocardiograms. Histograms showing summary data for mean fractional area change pre (−) and post (+) intravenous dobutamine (DOB) infusion in (**F**) young, (**G**) aged, control, and HF animals as indicated. **p < .01 vs Control pre-DOB; ***p < .001 vs Control pre-DOB; ^#^ *p* < .05 vs HF pre-DOB; ^$^ *p* < .05 vs Control post-DOB; ^$$$^ *p* < .001 vs Control post-DOB. (**H**) Summary data showing plasma noradrenaline concentrations determined by competitive ELISA. YC, young control; YF, young heart failure; OC, aged control; OF, aged heart failure. ^#^ *p* < .05 vs OC. For panels (**A**–**D**) and (**F**, **G**), *n* = 5 young, 5 aged, for panel (**E**) *n* = 7 young, 5 aged, and for panel (**H**), *n* = 9 young, 7 aged.

We next sought to determine the time course of development and extent of sympathetic dysfunction in aging and with the onset of HF. This was assessed weekly by determining the HR responses to dobutamine (5–40 µg·kg^−1^·minute^−1^, Figure 2A). Before the onset of tachypacing to induce HF, dobutamine (5 µg·kg^−1^·minute^−1^) increased HR in both young and aged animals by 63% ± 14% in young and 42% ± 14% in aged (Figure 2B and C; *p* < .001 vs pre-dobutamine; *p* = .148 between age groups). Although this dose of dobutamine was still sufficient to increase HR compared with baseline after 1 week (*p* < .01) and 3 weeks (*p* < .05) tachypacing in the young, this was not the case for aged animals where, after just 1 week of tachypacing, no change in HR was observed in response to 5 μg·kg^−1^·minute^−1^ dobutamine. We also noted that although there was a robust increase in HR in response to 5 μg·kg^−1^·minute^−1^ dobutamine in both young and aged animals prior to commencement of tachypacing, at the onset of HF, we found no change in HR response to doses of dobutamine less than 20 μg·kg^−1^·minute^−1^ (Figure 2D); thus, the chronotropic response to β-adrenergic stimulation is reduced in HF in both age groups although appears to occur earlier in the aged animals.

### Sympathetic-mediated Inotropic Function Is Attenuated With Aging and HF

We next assessed whether the inotropic response to β-adrenergic stimulation, assessed as LV fractional area change, is attenuated in aging and with the development of HF (Figure 2E–G). In both young and aged animals, cardiac contractility (fractional area change) decreased with the development of HF with no age-dependent temporal differences observed (Figure 2E). In young prepaced animals, 5 μg·kg^−1^·minute^−1^ dobutamine increased fractional area change by 31% ± 10%, whereas in aged control animals, this dose of dobutamine, despite its effect on HR, did not increase LV contractility significantly from baseline (*p* = .129). On development of HF, the contractility response to catecholamine stimulation was assessed with an elevated dose of dobutamine which led to an increase of HR to 150 bpm (“the effective dose”; 20 µg·kg^−1^·minute^−1^ in 8 of 10 animals; range 10–40 µg·kg^−1^·minute^−1^). In young HF animals, the effective dose of dobutamine (10–40 µg·kg^−1^·minute^−1^) increased contractility by 31% ± 13% (*p* < .05) and in aged HF sheep, the effective dose of dobutamine (20 µg·kg^−1^·minute^−1^) increased fractional area change by 29% ± 15% (*p* < .05). In both young and aged HF animals, despite the increased doses of dobutamine, the fractional area changes were considerably less than the 5 µg·kg^−1^·minute^−1^ dobutamine responses observed before tachypacing commenced (*p* < .05). The attenuated chronotropic and inotropic responses to dobutamine in HF were also associated with a significant interaction between HF and plasma noradrenaline levels increasing (Figure 2H; *p* < .05; two-way RM-ANOVA) with a directly observed increase in the aged cohort (aged control, 557±73 pg·mL^−1^; aged HF, 1973±937 pg·mL^−1^; *p* < .05).

### Parasympathetic Control of HR Is Lost With HF in Both Young and Aged Animals

**Figure 3.**
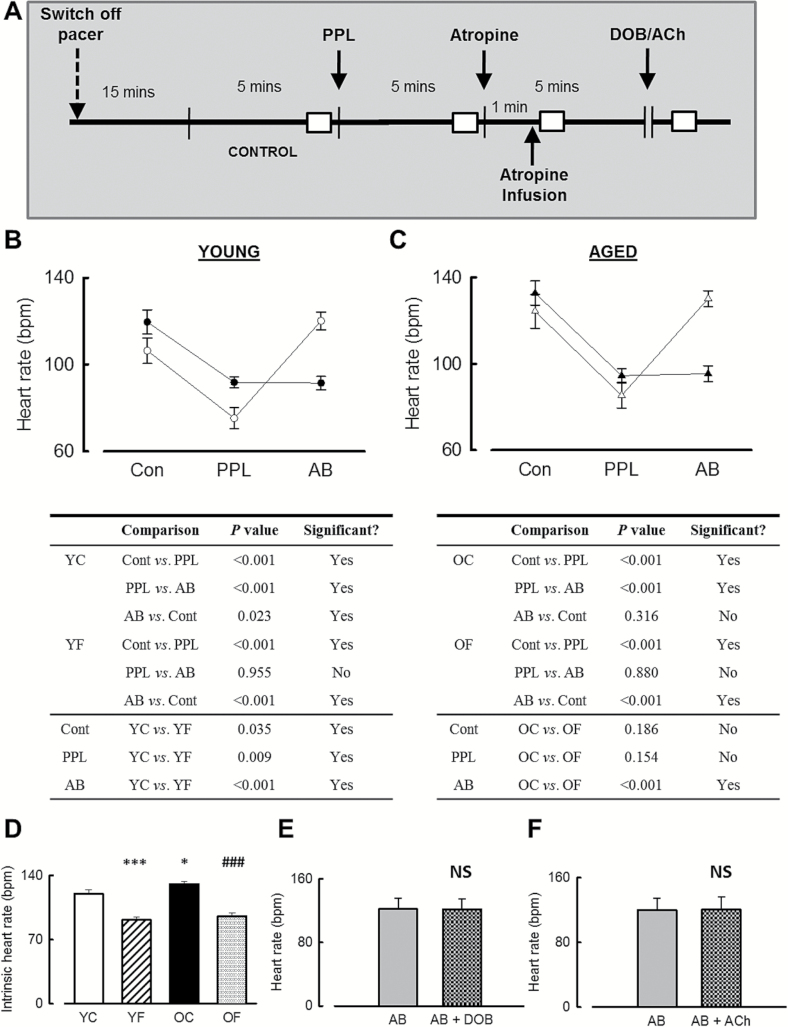
Heart failure and aging mediated parasympathetic dysfunction revealed by sequential autonomic blockade. (**A**) Schematic representation of experimental approach to achieve sympathetic and full autonomic blockade (AB). The white boxes indicate time periods used to analyze data. The horizontal break indicates assessment of responsiveness to dobutamine (DOB) or acetylcholine (ACh) following autonomic blockade performed in a subset of animals. (**B**) Summary data showing (upper panel) measured HR predrug administration (control, Con), after intravenous bolus of propranolol (PPL) and atropine (AB, full autonomic block) obtained prepacing (open symbols) and HF (closed symbols) in young animals and, (lower panel) tabulated significance values. (**C**) Summary data (upper panel) and tabulated significance data (lower panel) for aged animals (triangles, right). *n* = 13 young, 11 aged. (**D**) Mean intrinsic HR in aging and HF. *p < .05 vs YC; ***p < .001 vs YC; ^###^ *p* < .001 vs OC. *n* = 13 YC, 13 YF, 15 OC, 11 OF. (**E**, **F**). Absence of heart rate responses to dobutamine infusion (**E**) or acetylcholine (**F**) following autonomic blockade (AB). NS, not significant. *n* = 4 each group.

Assessment of parasympathetic control of HR and HRV was made by sequentially blocking sympathetic and parasympathetic input with PPL and atropine, respectively, to achieve full autonomic blockade (AB, Figure 3A).

In young prepaced animals (Figure 3B), β-adrenergic receptor antagonism with PPL decreased HR by 29% ± 6% (*p* < .001) and subsequent full autonomic blockade led to a 60% ± 12% increase in HR (*p* < .001, compared with PPL). The mean intrinsic HR (ie, outwith autonomic influence) of young prepaced animals was 13% ± 7% faster than the resting HR (predrug administration; *p* < .05). In young HF animals, PPL still decreased HR (by 20% ± 6%, *p* < .001). However, this was a smaller reduction in HR than that seen before tachypacing commenced (*p* < .01), suggesting reduced sympathetic influence in HF. Furthermore, in the young HF animals, subsequent autonomic blockade failed to have any additional effect on HR (*p* = .955) indicating the loss of parasympathetic influence on HR control.

A broadly similar pattern was observed within the aged group (Figure 3C), with PPL slowing HR by 31% ± 6% compared with control conditions (*p* < .001) and autonomic blockade increasing HR by 53% ± 11% compared with PPL (*p* < .001). However, unlike in the young control animals, the intrinsic HR of aged control animals, that is, during autonomic blockade, was not different to the aged control resting HR (*p* = .316). Furthermore, the intrinsic HR in aged control animals is greater than that in young control animals (young, 120.1±4.1 bpm; aged 130.8±3.0 bpm. Figure 3D, *p* < .05). In aged failing animals, PPL again slowed HR (by 29% ± 4%), but this was to a similar extent to that seen in the prepacing situation (*p* = .154). In aged HF animals, autonomic blockade failed to increase HR following PPL administration (*p* = .88). Thus HF in aged animals was associated with a loss of parasympathetic influence on HR, but sympathetic influence was unaltered.

The only age-dependent differences in HR noted were those present at baseline (ie, prior to sympathetic or parasympathetic blockade). In both prepaced and postpaced time points, aged sheep had a higher HR than in the young sheep (both *p* < .05).

Importantly, the dosages of PPL and atropine used to achieve autonomic blockade were effective at preventing any HR responses to subsequent doses of dobutamine or acetylcholine (Figure 3E and F).

### Parasympathetic Control of HRV Is Lost With the Development of HF

**Figure 4.**
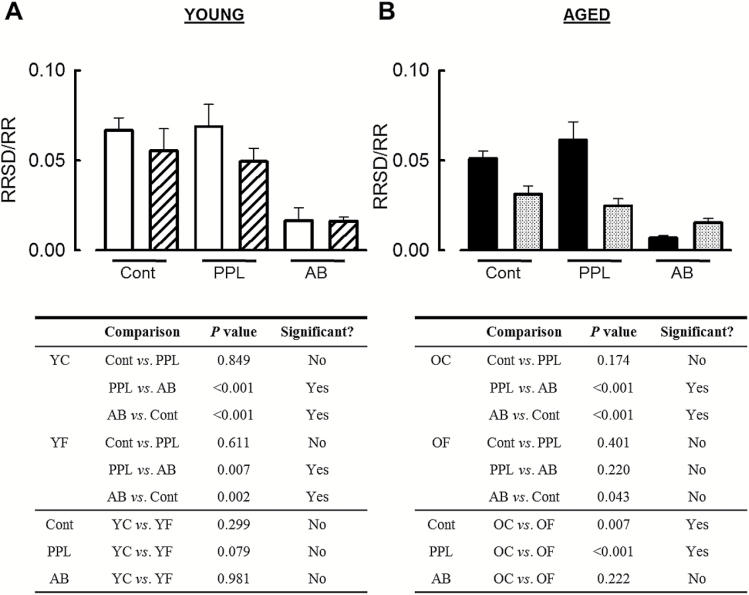
Aging and heart failure mediated reductions in heart rate variability. (**A**) Summary data (upper panels) showing RRSD covariance values predrug administration (control, con), after propranolol (PPL) and atropine (full autonomic blockade, AB) in young prepaced (open bars) and HF (striped bars) and (lower panels) table of significance values for young animals. (**B**) Data (upper panels) and summary of significance (lower panels) for aged control (solid bars) and HF (checked bars) animals. *n* = 13 young, 11 aged.

We also measured HRV from the RRSD/RR ratio following β-adrenergic receptor blockade and full autonomic blockade in young (Figure 4A) and aged (Figure 4B) animals. In young prepaced animals, no difference in HRV was detectable during β-adrenergic blockade with PPL, but HRV was reduced during full autonomic blockade (control, 0.067±0.007; PPL, 0.069±0.012; AB, 0.017±0.007. All comparisons *p* < .001). A similar trend in HRV was seen in young failing animals following sequential β-adrenergic and full autonomic blockade (control, 0.056±0.012; PPL, 0.049±0.007; AB, 0.016±0.002. All comparisons *p* < .01) such that, in young animals under full autonomic blockade, no differences in HRV exist between the prepaced and HF conditions.

As with the young prepaced sheep, no difference in HRV was found in aged prepaced animals following PPL, but HRV was less during full autonomic blockade (control, 0.051±0.004; PPL, 0.061±0.01; AB, 0.007±0.001. All comparisons *p* < .001). However, in aged failing animals, neither PPL nor full autonomic blockade altered the coefficient of variation of RRSD (RRSD/mean RR; control, 0.031±0.005; PPL, 0.025±0.004; AB, 0.015±0.002). The coefficient of variance of RRSD measure of HRV demonstrated that HRV was less at both baseline (no drug infusions) and following β-adrenergic blockade in aged failing sheep compared with the prepacing values (*p* < .01).

The only age-dependent difference in the coefficient of variance of RRSD measurement of HRV following sequential β-adrenergic antagonism and full autonomic blockade was in HF both at baseline and after PPL administration. Thus HRV was less in aged HF animals than in young HF animals at baseline (no drug infusion) and following β-adrenergic receptor antagonism (*p* < .05). Following full autonomic blockade, HRV (coefficient of variance of RRSD) was the same in both young and aged HF animals.

## Discussion

The aims of the present study were to determine whether aging influences the evolution of autonomic dysfunction during the development of HF. The major findings were (i) cardiac dimensions are greater and indices of contractility are less in aged HF animals; (ii) chronotropic and inotropic responses to β-adrenergic stimulation are reduced in both aged and young animals with HF but develop earlier in the progression of tachypacing-induced HF in the aged; (iii) time-domain measures of HRV are reduced as a consequence of HF in both young and aged animals, but these are more pronounced in the aged, with some indices showing reduced HRV as a consequence of aging alone; and (iv) following sequential autonomic blockade to assess the sympatho-vagal influence on the control of HR we found subtle age-dependent differences in HF, although before tachypacing commenced the intrinsic HR (ie, during full autonomic blockade) was greater than the resting HR in young animals. Together these findings indicate that there are subtle age-dependent effects on (i) the extent of cardiac remodeling, (ii) β-adrenergic responsiveness, and (iii) autonomic control of HR with the development of HF.

Aging is known to influence a number of cardiac physiological and structural variables including the occurrence of cardiac fibrosis (17,23,24), mitochondrial dysfunction, (25) and in the context of the present study, impaired inotropic reserve and exercise intolerance; the latter being considered hallmarks of aging (8). The opposing actions of the sympathetic and parasympathetic innervation of the heart ultimately determine HR and contribute to the beat-to-beat variability of HR, and these controlling factors are known to be altered by aging (26,27). We show that aging also influences these parameters in response to the development of HF. Specifically, using an ovine model of aging we show that HR is increased whereas time-domain measures of HRV and the inotropic response to β-adrenergic stimulation are reduced. These findings are consistent with a diminished responsiveness to catecholamines in aging being observed at the cellular (28,29), tissue (8), and whole heart (30) levels. The elevated plasma catecholamine levels noted in some studies of aging [(31,32); Figure 2H] may be directly contributing to the decreased inotropic and chronotropic adrenergic responsiveness of the aged heart due to desensitization and internalization of the β-adrenergic receptors as has been previously noted in both aging (11) and HF (4).

A substantial body of evidence demonstrates that the elevated sympathetic drive in HF ultimately leads to a diminished myocardial response to β-adrenergic stimulation, for example, see (3,4,33). In this study, we have demonstrated that the development of impaired β-adrenergic responsiveness occurs earlier in aged animals during the progression of HF. A similar age-dependent attenuation of β-adrenergic influence on HR has been noted following exercise in patients (34). However, although the aged HF animals develop impaired inotropic and chronotropic responses to β-adrenergic stimulation earlier than the young HF animals, there are no age-dependent difference in β-adrenergic responsiveness once HF has developed, that is, HF results in a marked attenuation of the chronotropic response in both the young and aged. Thus aging may be an important factor determining the temporal development, rather than ultimate outcome, of, impaired responsiveness to catecholamines as a result of pathological stresses such as HF.

Although we were unable to monitor the temporal development of impaired parasympathetic influence on HR control with the onset of tachypacing, we did note that, in both young and aged animals, there was a marked loss of the parasympathetic control of HR once HF had developed. Classically, the parasympathetic influence on the heart is to slow sinus node firing rate and thus HR. Additionally, the parasympathetic input to the heart is also thought to determine the short-term variability of HR, albeit as a saturating function of parasympathetic activity (35,36). The increase in resting HR with age, as well as the unaltered HR following full autonomic block noted in aged animals, suggests that there is a reduced parasympathetic component to the control of HR in aging. Moreover, following tachypacing, there is a dramatic reduction/loss of parasympathetic input to controlling HR in both age groups, as demonstrated by (i) the marked reductions in several time-domain measures of HRV and (ii) the failure of parasympathetic blockade with atropine to influence HR following β-adrenergic blockade with PPL. Such observations are common to numerous studies of HF. However, the influence of aging on this response has been less well characterized (37); albeit that, at least in the present study, aging does not appear to influence this outcome once HF has developed. Given that reduced parasympathetic activity is generally held to indicate poor outcomes in HF, for example, see (38), the beneficial effects of chronic vagal nerve stimulation in HF may, in part, arise from the improved autonomic control of HR (6). Further studies are required to elucidate other cardioprotective mechanisms that may arise from vagal nerve stimulation.

### Study Limitations

Although the tachypacing approaches are considered useful models of dilated cardiomyopathy and tachycardic cardiomyopathy (39), they do not replicate the most common cause of HF occurring as a result of myocardial ischemia. However, the autonomic perturbations noted in the sheep model of aging and tachypacing-induced HF are consistent with those seen in other causes of HF and suggest a common effect on the sympatho-vagal control of HR independent of the underlying cause of this condition. Secondly, we did not perform weekly assessments of parasympathetic function (using autonomic blockade), during the development of HF due to the risk of arrhythmias once tachypacing was resumed while animals were still subject to the effects of circulating PPL and atropine. However, the chronotropic response to β-adrenergic stimulation was assessed weekly and demonstrated that sympathetic dysfunction arises very early in the development of HF. Finally, due to the orientation of the cardiac apex over the sternum, we were unable to obtain conventional transthoracic long-axis apical views of the heart by echocardiography and are therefore unable to satisfactorily determine a number of indices of cardiac function, for example, *E*/*A* ratios, torsion, and ejection fraction. Nevertheless, the long- and short-axis views that we have quantified clearly show a marked deterioration in cardiac function with aging and the development of HF.

## Conclusion

In conclusion, we have demonstrated that aging results in cardiac remodeling, reduced contractility, reduced HRV assessed by some time-domain analysis approaches, and reduced response to β-adrenergic stimulation. With the development of HF, we noted that aging is associated with a more rapid loss of the chronotropic response to β-adrenergic stimulation, greater cardiac dimensions, and impaired contractility, and finally, subtle changes in sympatho-vagal imbalance compared with young HF animals. Taken together, given the age dependence of the development of HF in the general population, our findings suggest that aging is an important factor determining the development and extent of autonomic dysfunction in HF. The outcomes of current clinical trials targeting the vagal nerve may therefore be influenced by patient age.

## Funding

This work was supported by grants from The British Heart Foundation (FS/12/57/29717, FS/09/036, PG/11/16, PG/10/89, PG/09/062, and FS/07/003).

## Conflict of Interest

None.
